# Changes in brain activity of somatoform disorder patients during emotional empathy after multimodal psychodynamic psychotherapy

**DOI:** 10.3389/fnhum.2013.00410

**Published:** 2013-08-16

**Authors:** Moritz de Greck, Annette F. Bölter, Lisa Lehmann, Cornelia Ulrich, Eva Stockum, Björn Enzi, Thilo Hoffmann, Claus Tempelmann, Manfred Beutel, Jörg Frommer, Georg Northoff

**Affiliations:** ^1^Department of Psychosomatic Medicine and Psychotherapy, University Medicine MainzMainz, Germany; ^2^Department of Psychosomatic Medicine and Psychotherapy, University Hospital LeipzigLeipzig, Germany; ^3^Department of Psychosomatic Medicine and Psychotherapy, Otto-von-Guericke-University HospitalMagdeburg, Germany; ^4^Department of Cardiology, Klinikum BielefeldBielefeld, Germany; ^5^Department of Psychotherapeutic Medicine, Fachklinikum UchtspringeUchtspringe, Germany; ^6^Department of Psychiatry, Ruhr-University Bochum, LWL University HospitalBochum, Germany; ^7^Department of Psychotherapeutic Medicine, Diakoniewerk HalleHalle, Germany; ^8^Department of Neurology, Otto-von-Guericke University HospitalMagdeburg, Germany; ^9^Mind, Brain Imaging and Neuroethics Unit, Institute of Mental Health Research, University of OttawaOttawa, ON, Canada

**Keywords:** psychodynamic, psychotherapy, fMRI, emotional empathy, somatoform disorder

## Abstract

Somatoform disorder patients show a variety of emotional disturbances including impaired emotion recognition and increased empathic distress. In a previous paper, our group showed that several brain regions involved in emotional processing, such as the parahippocampal gyrus and other regions, were less activated in pre-treatment somatoform disorder patients (compared to healthy controls) during an empathy task. Since the parahippocampal gyrus is involved in emotional memory, its decreased activation might reflect the repression of emotional memories (which—according to psychoanalytical concepts—plays an important role in somatoform disorder). Psychodynamic psychotherapy aims at increasing the understanding of emotional conflicts as well as uncovering repressed emotions. We were interested, whether brain activity in the parahippocampal gyrus normalized after (inpatient) multimodal psychodynamic psychotherapy. Using fMRI, subjects were scanned while they shared the emotional states of presented facial stimuli expressing anger, disgust, joy, and a neutral expression; distorted stimuli with unrecognizable content served as control condition. 15 somatoform disorder patients were scanned twice, pre and post multimodal psychodynamic psychotherapy; in addition, 15 age-matched healthy control subjects were investigated. Effects of psychotherapy on hemodynamic responses were analyzed implementing two approaches: (1) an a priori region of interest approach and (2) a voxelwise whole brain analysis. Both analyses revealed increased hemodynamic responses in the left and right parahippocampal gyrus (and other regions) after multimodal psychotherapy in the contrast “empathy with anger”—“control.” Our results are in line with psychoanalytical concepts about somatoform disorder. They suggest the parahippocampal gyrus is crucially involved in the neurobiological mechanisms which underly the emotional deficits of somatoform disorder patients.

## Introduction

Somatoform disorders contain a group of complex diseases consisting of medically unexplained somatic symptoms (Kirmayer et al., 1994; Stein and Muller, 2008; Pedrosa Gil et al., 2009; Hiller et al., 2010).

Psychologically, they are linked to alexithymia, a construct which describes decreased emotional awareness (Sifneos, 1973; Bach and Bach, 1996; Bankier et al., 2001; Duddu et al., 2003; Grabe et al., 2004; Burba et al., 2006; Bailey and Henry, 2007; Mattila et al., 2008; Pedrosa Gil et al., 2008; Wood et al., 2009). In addition, another emotional process—emotion recognition (i.e., the correct labeling of emotions, Pedrosa Gil et al. 2009)—is impaired (Pedrosa Gil et al., 2009; de Greck et al., 2012), and somatoform disorder patients describe increased “empathic distress” (i.e., they experience themselves as being easily affected and overwhelmed by negative emotional states of others, Davis 1983; de Greck et al. 2012).

From a psychodynamic perspective, emotional alterations of somatoform patients are interpreted as being caused by the unconscious repression of specific emotions to avoid interpersonal conflicts, which would cause strong negative affects (Bowlby, 1973; Waller and Scheidt, 2006). Somatizing patients are thus unable to verbally express their emotional states whilst they still experience the somatic component related to their affective reaction (as symptom, Schur, 1955; Krystal, 1997; Beutel et al., 2008). In addition, increased attention to body sensations (in order to distract from interpersonal conflicts) plays an important role (Eriksen and Ursin, 2004; Nakao and Barsky, 2007; Witthöft and Hiller, 2010; de Greck et al., 2011).

Neurophysiologically, as was demonstrated in a previous paper by our group, pre-treatment somatoform disorder patients show diminished modulation of neuronal activity in several brain regions including the bilateral parahippocampal gyrus, the left amygdala, the left postcentral gyrus, the left superior temporal gyrus, and the left posterior insula, during emotional empathy (compared to healthy control subjects, de Greck et al. 2012). In particular, diminished neuronal activation of the parahippocampal gyrus is highly interesting, since other studies emphasized the crucial role of this region in the recall of autobiographical memories (Maguire, 2001; Niki and Luo, 2002; Rekkas and Constable, 2005; Beni and Venneri, 2006). Especially the retrieval of emotional memories activates the parahippocampal gyrus: For instance, Damasio and colleagues showed that the parahippocampal gyrus is involved in the induction of emotion by intentional retrieval of autobiographic emotional memory (Damasio et al., 2000). In addition, Smith and colleagues (2004) and Sterpenich and colleagues (2006) showed that the parahippocampal gyrus is also involved in the retrieval of emotional background contexts during the active recall of memorized neutral stimuli. Even more interesting, two studies found evidence showing that the parahippocampal gyrus is particularly involved in the processing of conflict related memories: Loughead and colleagues (2010) investigated brain activity during the recall of autobiographic relationship episodes, while also checking for relationship conflict. They found that activity in the parahippocampal gyrus was positively correlated with the degree of conflict related to autobiographical episodes. In complement to this, Schmeing and colleagues (2013) reported a deactivation of the parahippocampal cortex during free associations to conflict-related sentences (when compared to neutral sentences). Subsequent to the free association task, the authors included an unexpected memory recall task. They found that free associations to conflict-related sentences were more often forgotten when compared to free associations made to neutral sentences.

The diminished activation of the parahippocampal gyrus found during emotional empathy in somatoform disorder patients might hence reflect the disturbed retrieval of repressed emotional memories; accordingly, it might be a neurobiological correlate of repression.

Psychodynamic psychotherapy is an established therapy in the treatment of somatoform disorder (and other mental disorders, Leichsenring 2005; Gemeinsamer Bundesausschuss 2009). In the treatment of somatoform disorders, psychodynamic psychotherapy aims to increase the insight and acceptance of unconscious needs and emotional conflicts which underly the client's symptoms (Blagys and Hilsenroth, 2002; Leichsenring, 2005); thus, interpretations are a key instrument (Crits-Christoph et al., 1988). The process of “working through” aims to enable patients to utilize other (namely “healthier”) coping strategies (Vaillant, 1977; Wöller and Kruse, 2010), leading to less somatic symptoms and a more satisfying life-style. Further aims of psychodynamic psychotherapy in somatoform disorder include enhancement of the mentalization function, improvement of affect perception and the reduction of medication abuse (Beutel et al., 2008).

Aim of this study was to investigate whether neuronal activity in the parahippocampal gyrus normalized after psychotherapy (mediated by the uncovering of repressed emotional memories). In addition, we were also interested, whether any of the other regions with diminished neuronal activity in the pre-treatment stage (i.e. the left amygdala, the left postcentral gyrus, the left superior temporal gyrus, and the left posterior insula) showed a normalization of neuronal activity in the post-treatment stage.

## Methods

### Ethical approval

The study was ethically approved by the Institutional Review Board of the Otto-von-Guericke University of Magdeburg/Germany. After a detailed explanation of the study, all subjects gave informed consent. All subjects received financial compensation for their participation in the study. The study was conducted at the Otto-von-Guericke University of Magdeburg/Germany.

### Participants

We investigated 15 patients (8 females, 7 males; 14 right handed, 1 left handed; mean age: 42.6 years, 95%-confidence interval: 35.0–50.1 years) suffering from a somatoform disorder as ascertained by the Structured Clinical Interview for DSM-IV (German version: SKID, Wittchen et al. 1997). 11 of the 15 patients fulfilled criteria of an undifferentiated somatoform disorder (DSM-IV: 300.82), 2 of the 15 patients had a somatization disorder (DSM-IV: 300.81), and 2 of the 15 patients had a pain disorder (DSM-IV: 307.80). Leading symptoms of the pre-treatment patients included different forms of pain (e.g., back pain, neck pain, headache), abdominal disturbances (e.g., diarrhea, flatulence, abdominal pain), sexual dysfunctions, and others. All patients were recruited at the start of an inpatient psychotherapy. Patients were recruited from the Department of Psychosomatic Medicine and Psychotherapy of the Otto-von-Guericke-University Hospital in Magdeburg (9/15), from the Department of Psychotherapeutic Medicine of the Fachklinikum Uchtspringe (3/15), and from the Department of Psychosomatic Medicine and Psychotherapy of the AWO Hospital Jerichow (3/15). All patients underwent a second fMRI session at the end of their psychotherapy. The time difference between both scanning session was 58 days on average (95%-CI: 51–65 days; range: 38–80 days). During the first fMRI session, 5 patients were on psychotropic medication with duloxetine. During the second fMRI session, one patient continued with duloxetine and one other patient continued with duloxetine and trimipramine. In addition to the patient group, we also investigated 15 gender matched and age matched healthy control subjects (8 females, 7 males; 12 right handed, 1 left handed, 2 ambidextrous; mean age: 37.0 years, 95%CI: 34.4–45.4 years, *t*_(28)_ = 0.614; *p*_[two−tailed_] = 0.545). This study included 15 patients, who were scanned in their pre-treatment stage and in their post-treatment stage. These 15 patients were taken out of a larger population of 20 patients who were scanned in in the pre-treatment stage. Due to different reasons, however, 5 of those 20 patients were not scanned in their post-treatment stage. Drop out reasons included premature termination of psychotherapy (3/20), refusal to participate a second time (1/20), or inaccessibility after discharge (1/20).

In addition, this study is part of a larger trial where we compared patients with somatoform disorder patients pre and post multimodal inpatient psychodynamic treatment and healthy control subjects. Data obtained in this trial have already been presented in two previous papers of our group: In one study, we investigated 20 pre-treatment somatoform patients and 20 healthy subjects using the same paradigm (de Greck et al., 2012). In another study, we investigated 20 pre-treatment somatoform patients, 15 post-treatment somatoform patients, and 20 healthy subjects using a different paradigm (de Greck et al., 2011). In the here presented study, we focus on the effects of multimodal inpatient psychodynamic treatment on brain activity of somatoform disorder patients during emotional empathy—these data have not been published before.

### Psychotherapeutic intervention

All patients participated in a standardized inpatient multimodal psychodynamic psychotherapy (henceforth “psychotherapy”), which was conducted as recently explained (Grabe et al., 2008; Haase et al., 2008; Huber et al., 2009; de Greck et al., 2011). The therapeutic setting was multimodal and included psychodynamic individual therapy, psychodynamic group therapy, medical therapy, and other therapeutic methods including music therapy, communicative movement therapy, art therapy, social therapy, and relaxation methods (see the Supplementary Material for a more detailed explanation of the therapeutic techniques). Psychotherapy aimed to improve the verbalization of emotional and interpersonal problems, to improve affect perception, and to enhance the understanding of intra-psychic and interpersonal conflicts underlying the patient's symptoms. (Leichsenring, 2005; Beutel et al., 2008; Grabe et al., 2008). Thereby, psychotherapy aimed to enable the patient to utilize a broader spectrum of coping strategies (Vaillant, 1977).

### Psychological scales

Several psychological measurements were used to investigate differences between patients and healthy subjects as well as differences between patients in the pre-treatment and post-treatment stage. Psychological data of healthy subjects were assessed only once.

Somatization was assessed by the respective sub-scale of the “Symptom Check List 90 - Revised Version” (SCL-90-R, German edition, Derogatis, 1977; Franke, 2002), a self-report questionnaire, which contains several sub-scales. SCL-90 somatization scores were collected from 15 of the 15 pre-treatment somatoform patients, 12 of the 15 post-treatment somatoform patients, and 14 of the 15 healthy control subjects.

Emotional awareness was tested by the German version of the well established self-report questionnaire “Toronto Alexithymia Scale - 20” (TAS-20, Bagby et al., 1994; Bressi et al., 1996). TAS-20 scores were collected from the 15 pre-treatment somatoform patients, the 15 post-treatment somatoform patients, and the 15 healthy control subjects.

Mood state and in particular depressive symptoms were assessed with a German edition of the “Beck Depression Inventory” (BDI, Beck et al., 1961). BDI scores were ascertained from the 15 pre-treatment somatoform patients, the 15 post-treatment somatoform patients, and the 15 healthy control subjects.

Emotion recognition abilities were tested using the “Tübinger Affekt Batterie” (TAB, Breitenstein et al. 1998), the German version of the “Florida Affect Battery” (FAB, Bowers et al., 1999). We applied four sub-tests of the TAB: TAB3 and TAB5, which use emotional face stimuli, TAB8a, which uses spoken emotional sentences to test for the ability to identify prosody and semantic content, and TAB8b, which uses spoken sentences as the TAB8a, but applies a number of incongruent auditory stimuli (i.e., sentences with different prosodic and emotional content). The average error rate of all sub-tests was included into the analysis. TAB scores were obtained from 15 pre-treatment somatoform patients, 14 post-treatment somatoform patients, and 12 healthy control subjects.

Statistical analyses of psychological scales included paired samples t-tests to investigate potential effects of psychotherapy, and Spearman-correlations to check for correlations between different scales. Spearman correlations (and not Pearson correlations) were used with regard to the non-linear characteristics of the different scales. We implemented one-tailed tests if we had a directed a priori hypothesis (e.g., improvement of symptom scores in the post-treatment stage), and two-tailed tests otherwise (e.g., differences between two conditions).

### Paradigm

The paradigm contained a combination of two tasks, a reward anticipation task and an empathy task, which were separated from each other in a block wise manner. Here we report only results obtained from the empathy blocks; please, see our previous paper for results obtained during the reward anticipation paradigm (de Greck et al., 2011).

### Experimental design

Please also refer to our previous paper for an in depth description (de Greck et al., 2012). Subjects read detailed information about the paradigm and completed a couple of trial runs in order to familiarize with the experiment. In the scanner, stimuli were projected onto a matt screen via an LCD projector, which was visible through a mirror mounted on the head coil. During the *experiment* three empathy blocks were presented. Each *block* started with a short finger tapping task. Directly afterwards the actual empathy session began with the presentation of a short instruction, which lasted for 6 s. A total number of 40 empathy trials were then presented in a random order. After every 8 empathy trials, a short pause occurred, lasting for 6, 7, or 8 s duration; during pauses, the fixation cross was presented. At the end of each block, subjects were asked to rate their present feeling for contentedness as well as their impression of engagement in the empathy task, by moving a bar on a visual analogue scale. Each *trial* began with the display of an emotional face or a control stimulus lasting for 5 s. Subjects were instructed to empathize with the presented emotional face, which was expressed by the phrase “please try to share the emotional state of the person shown.” Immediately after the presentation of the emotional face, subjects were asked to rate their ability to empathize with the preceding picture by moving a bar of a visual analogue scale. Prior to the following empathy trial a short inter trial interval (lasting for 2 or 3 s) was presented. Facial stimuli expressing the emotion conditions anger, disgust, joy, and neutral emotional state were implemented. Smoothed pictures with unrecognizable contents served as control stimuli.

### Stimuli

The emotional face stimuli were taken from two batteries: the “Japanese and Caucasian Facial Expressions of Emotion”-battery (JACFEE) and the “Japanese and Caucasian Neutral Faces”-battery (JACNeuF), both provided by Matsumoto and Ekman (Matsumuto and Ekman, 1988). Eight different facial stimuli of every emotion condition (anger, disgust, joy and neutral) were shown, resulting in 32 different stimuli. Stimuli depicted 16 Caucasian and 16 Japanese actors, half of them female, half of them male. 8 smoothed pictures with unrecognizable contents served as control stimuli. Subjects were instructed to rate the smallest empathy amount (zero) after a control stimulus was presented. During the whole experiment each stimulus was presented once in each block, and for three times during the entire experiment.

### Functional magnetic resonance imaging (fMRI)

#### fMRI data collection

fMRI data were collected in a 1.5T MR scanner (General Electric Sigma Horizon) using a standard circular polarized head coil. A stack of 23 slices was aligned parallel to the bicomissural plane. During functional runs 320 whole brain volumes were acquired (gradient echo EPI, *TR* = 2 s; *TE* = 35 ms; flip angle α = 90°; Field of View = 200 × 200 mm; slice thickness = 5 mm, inter-slice gap = 1 mm, spatial resolution = 3.125 × 3.125 × 5 mm). Additionally, a T1 weighted image of every subject was acquired (3D-FSPGR, 60 saggital slices; *TR* = 8.8 ms; *TE* = 1.84 ms; flip angle α = 30°; Field of View = 230 × 173 mm; slice thickness = 2.8 mm, spatial resolution = 2.8 × 0.898 × 0.898 mm.

#### fMRI data analysis

Image processing and statistical analyses were carried out using the software package AFNI (http://afni.nimh.nih.gov/afni/, Cox 1996). The first five volumes of each functional run were discarded due to saturation effects. All functional images were slice-time corrected with reference to the acquisition time of the first slice and corrected for motion artifacts by realignment to the first volume. The images were spatially normalized to an AFNI-standard-EPI-template (“TT_EPI”) and re-sampled to 3 × 3 × 3 mm. Finally, all functional images were smoothed with an isotropic 6 mm full-width half maximum Gaussian kernel. T1-weighted images were normalized to a standard T1-template provided by AFNI (“TT_avg152T1”). For each subject, a voxelwise whole brain analysis was implemented, and regressors of interest were calculated by the convolution of a gamma response function with the according stimulus time functions (Josephs et al., 1997). All relevant periods (i.e., empathy periods, evaluation periods, pauses, and the free interval at the end of each session) were included in the model. In addition, six movement parameters resulting from the motion correction procedure were included as regressors to account for head motion effects. Likewise, nine regressors for the 3rd degree polynomial model of the baseline of each block were included to control for baseline fluctuations. Contrast images were calculated for each subject by employing linear contrasts to the parameter estimates for the regressors of each event (Friston et al., 1995).

This was followed by a second level group statistic, based on two approaches:

Firstly, we performed a statistical analysis of parameter estimates extracted from regions of interest (ROIs). ROIs were taken from a previous paper of our group in which we used the same paradigm (and partially the same subjects, see above) and found diminished modulation of brain activity of pre-treatment somatoform patients in several brain areas (de Greck et al., 2012). When compared to healthy controls, pre-treatment somatoform patients had shown diminished modulation in their hemodynamic responses in 12 regions (i.e., two regions for the contrast [“anger” + “disgust” + “joy” + “neutral expression”] − “control”, seven regions for the contrast “anger” − “control”, and three regions for the contrast “joy” − “control”). Spherical ROIs (with a radius of 5 mm) were defined based on the coordinates of those 12 regions, and mean contrast values of the according contrasts were extracted. Paired samples *t*-tests were implemented to check for significant differences between pre-treatment and post-treatment somatoform patients. With regard to the high number (12) of statistical tests, we applied a Bonferroni-correction to account for the multiple testing problem. Only those statistical results with a *p*-value of less than 0.05/12 = 0.004 were treated as significant results.

Secondly, we implemented a voxelwise second level random-effects analysis using paired samples *t*-tests (comparing the 15 somatoform patients in their pre-treatment stage and post-treatment stage) to identify brain regions which showed altered hemodynamic responses after psychotherapy. Again, we were only interested in contrasts, which had revealed a significant difference between pre-treatment somatoform patients and healthy control subjects in our previous study (de Greck et al., 2012) (namely [“anger” + “disgust” + “joy” + “neutral expression”] − “control,” “anger” − “control,” “joy” − “control”), whereas we did not investigate changes of hemodynamic responses after psychotherapy for the two other contrasts (namely “disgust” − “control” and “neutral expression” − “control”). To control for the multiple testing problem (Nichols and Hayasaka, 2003), we calculated family-wise error probabilities based on Monte-Carlo-simulations; in addition, small clusters with a size of ≤10 voxels were not respected. The anatomical localization and labeling of significant activations were assessed with reference to the standard stereotactic atlas of Talairach and Tournoux (1988) and by superimposition of the group contrast images on a mean brain generated by an average of normalized T1-weighted image of all patients.

Since 5 of the 15 patients were on psychotropic medication during either one or both scanning session, we implemented additional fMRI analyses including only data of those 10 participants who were without medication.

To check whether psychotherapy-induced reductions of somatic symptoms were associated with changes of hemodynamic responses in any of our regions of interest (i.e., the regions mentioned above plus significant regions of the voxelwise second level analysis), we implemented Spearman correlations of psychotherapy induced changes in SCL-90-somatization scores and psychotherapy induced changes of contrast values in the ROIs. We decided to implement Spearman correlations (and not Pearson correlations) with regard to the non-linear characteristics of the SCL-90-somatization scale.

Since we presented differences between pre-treatment somatoform patients and healthy subjects in a previous paper (de Greck et al., 2012), we here solely focused on the differences between pre-treatment and post-treatment somatoform patients. Data of healthy subjects are presented nevertheless for illustration purposes.

## Results

### Behavioral results

#### Psychological scales

**Figure 1:**
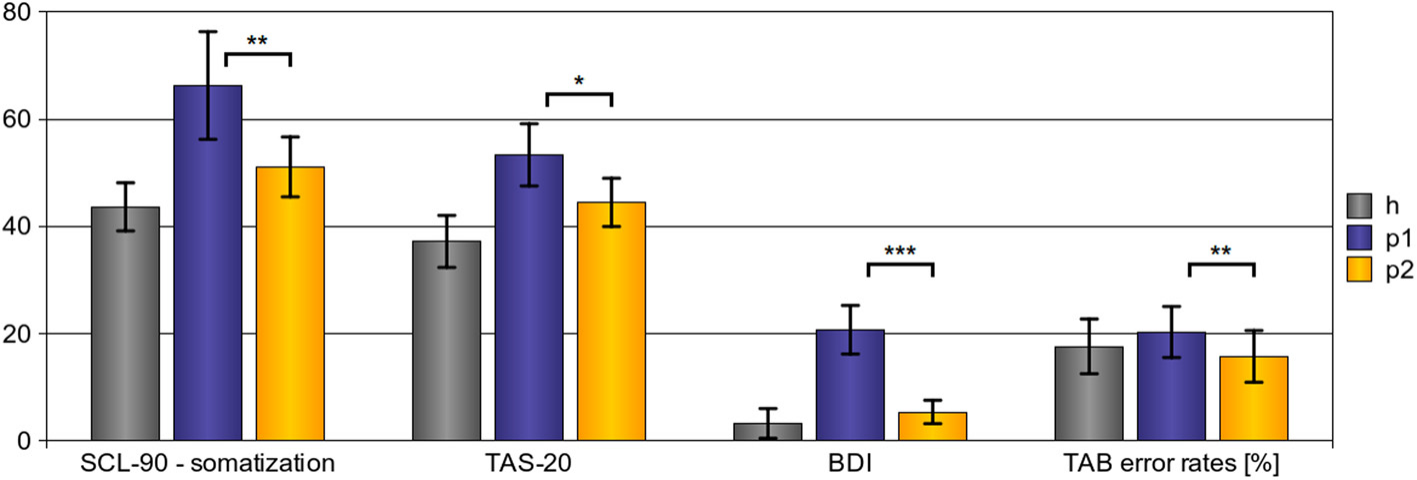
**Behavioral results—effects of psychotherapy.** After psychotherapy we observed a significant reduction of somatization severity (SCL-90 - somatization scores), to enhanced emotional awareness (TAS-20 scores), and to a reduction of depressive symptoms (BDI scores). In addition, emotion recognition abilities improved after psychotherapy as shown by a significant reduction of error rates in the TAB. (Explanations: *h*, *p*1, and *p*2 refer to the scores of healthy subjects (h), pre-treatment somatoform patients (*p*1), and post-treatment somatoform patients (*p*2); error bars indicate the 95%-confidence-interval; ^*^p < 0.05, ^**^p < 0.01, ^***^p < 0.001, with regard to one-tailed *t*-tests.)

As shown in Figure 1, psychotherapy had a significant effect on all applied outcome scales. *Somatization*, as assessed with the somatization sub-scale of the SCL-90-R, was significantly reduced after psychotherapy [*t*_(11)_ = 3.564; *p*_[one − tailed]_ = 0.002^**^]. *Emotional awareness*, as assessed with the TAS-20, was significantly enhanced after psychotherapy [*t*_(14)_ = 2.456; *p*_[one − tailed]_ = 0.014^*^]. In addition, *Mood state* was controlled using the BDI. After psychotherapy, we found a significant reduction of depressive symptoms [*t*_(14)_ = 5.660; *p*_[one − tailed]_ = 0.001^***^]. Finally, *emotion recognition abilities*, which were assessed using the TAB, improved; error rates were significantly lower after psychotherapy [*t*_(13)_ = 2.747; *p*_[one − tailed]_ = 0.008^*^].

When checking for correlations between different scales, we found that psychotherapy-induced reductions of SCL-90-somatization scores correlated with reductions of BDI-scores (ρ_[Spearman]_ = 0.558; *p*_[one − tailed]_ = 0.025^*^). In addition, psychotherapy induced reductions of TAS-20 scores correlated with reductions of BDI-scores (ρ_[Spearman]_ = 0.723; *p*_[one − tailed]_ = 0.001^**^). However, we did not find correlations of psychotherapy induced changes of SCL-90-somatization scores with changes in the TAS-20 (ρ_[Spearman]_ = 0.323; *p*_[one − tailed]_ = 0.306), of SCL-90-somatization changes with changes of error rates in the TAB (ρ_[Spearman]_ = −0.145; *p*_[one − tailed]_ = 0.673), of TAS-20 changes with changes of error rates in the TAB (ρ_[Spearman]_ = −0.123; *p*_[one − tailed]_ = 0.674), or (ρ_[Spearman]_ = 0.139; *p*_[one − tailed]_ = 0.637).

#### Intra-scanner empathy ratings

The 2 × 4 factorial ANOVA with “psychotherapy” (“pre-treatment” vs. “post-treatment”) as first within-subjects factor and “emotion” (“anger,” “disgust,” “joy,” and “neutral expression”) as second within-subjects factor revealed a significant effect of “emotion” [*F*_(3, 112)_ = 13.367; *p* < 0.001^***^], whilst we did not find significant effects for the factor “psychotherapy” [*F*_(1, 112)_ = 1.142; *p* = 0.288] or the interaction of “psychotherapy” × “emotion” [*F*_(3, 112)_ = 0.586; *p* = 0.626]. *Post-hoc t*-tests revealed, that empathy ratings for “anger” trials were significantly higher compared to “disgust” trials [*t*_(14)_ = 4.715; *p*_[two − tailed]_ < 0.001^***^] and significantly lower compared to “joy” trials [*t*_(14)_ = 5.924; *p*_[two − tailed]_ < 0.001^*^]. In addition, empathy ratings for “disgust” trials were significantly lower compared to “joy” trials [*t*_(14)_ = 7.880; *p*_[two − tailed]_ < 0.001^***^], and empathy ratings for “neutral expression” trials were significantly lower compared to “joy” trials [*t*_(14)_ = 6.479; *p*_[two − tailed]_ < 0.001^***^]. There were, however, no significant differences between empathy ratings for “anger” and “neutral expression” [*t*_(14)_ = 0.234; *p*_[two − tailed]_ = 0.818], and between empathy ratings for “disgust” and “neutral expression” [*t*_(14)_ = 1.621; *p*_[two − tailed]_ = 0.127].

### fMRI results

#### ROI based comparison of hemodynamic responses

In a previous paper, we found 12 brain areas (regions of interest, ROIs) with diminished modulation of hemodynamic responses in pre-treatment somatoform patients compared to healthy control subjects (de Greck et al., 2012). As shown in Table 2, we found significant improvement of hemodynamic responses after psychotherapy in 6 of the 12 regions—all for the contrast “anger”—“control.”

#### Voxel-wise whole brain analysis

**Figure 2:**
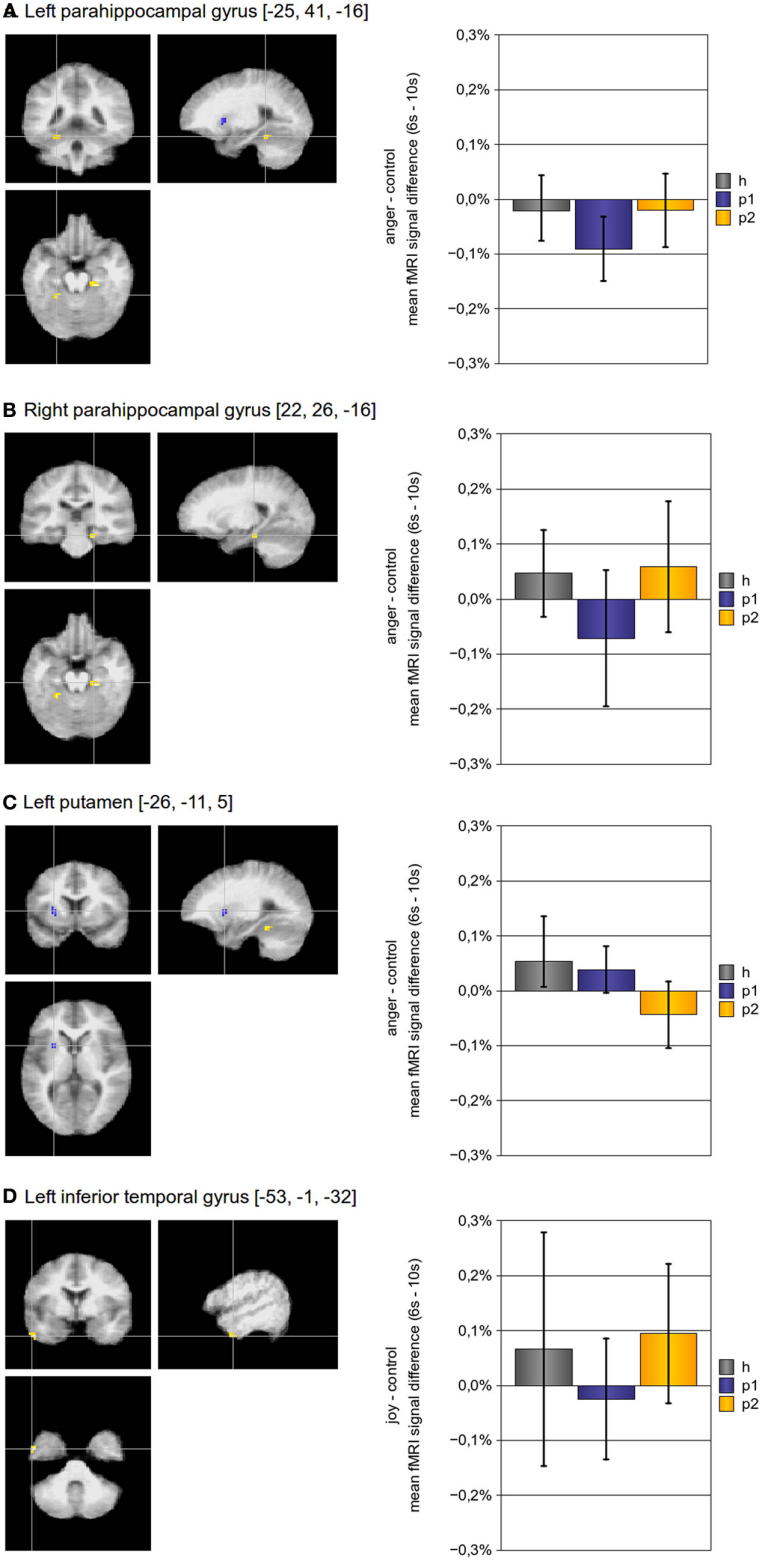
**Brain activity modulated by multimodal psychotherapy.** Using a voxel-wise whole brain analysis, we found three regions for the contrast “anger”-“control” and one for the contrast “joy”-“control”, which had a significant increase (bilateral parahippocampal gyrus, left inferior frontal cortex) or decrease (left putamen) of hemodynamic modulation after psychotherapy. The *p*-threshold was set to *p*_[uncorrected]_ ≤ 0.001; only clusters with a cluster size of more than 10 voxels were taken into account. (Abbreviations: *h*, *p*1, and *p*2 refer to the mean fMRI signal difference of the according contrast, where *h* indicates data of healthy subjects, *p*1 indicates data of pre-treatment somatoform patients, and *p*2 indicates data of post-treatment somatoform patients; error bars indicate the 95%-confidence-interval).

In addition to the above described ROI based approach, we also implemented a voxel-wise whole brain statistical analysis to identify brain regions with altered hemodynamic responses. As presented in Table 3 and Figure 2, four regions showed a significant effect of psychotherapy on hemodynamic modulation: The bilateral parahippocampal gyrus and the left inferior temporal gyrus showed increased modulation of hemodynamic responses after psychotherapy, the left putamen had diminished modulation of hemodynamic responses after psychotherapy.

#### Correlation of psychotherapy induced effects

We were interested, whether psychotherapy induced alleviations of somatic symptoms (as ascertained with the SCL-90-somatization sub-scale) correlated with psychotherapy induced changes of hemodynamic responses in any of our ROIs. With regard to those ROIs which had previously shown diminished modulation of hemodynamic responses in pre-treatment somatoform patients (i.e., ROIs listed in Table 1), we did not find any significant correlations under reasonable statistical thresholds (Spearman correlations, *p*_[two − tailed]_ < 0.05). With regard to the ROIs found in the voxel-wise analysis (i.e., ROIs listed in Table 2), we found only one significant correlation: the reduction in the SCL-90 somatization scores induced by psychotherapy correlated with the reduction of hemodynamic responses in the left putamen (ρ = 0.811; *p*_[one − tailed]_ = 0.001^**^).

**Table 1:** **Intra-scanner empathy ratings**.

**Condition**	***h***	***p*1**	***p*2**
Anger	62.6 ± 10.8	62.8 ± 11.8	63.9 ± 10.5
Disgust	61.2 ± 12.1	56.9 ± 12.2	56.8 ± 10.2
Joy	82.8 ± 8.5	86.7 ± 5.3	81.3 ± 7.6
Neutral expression	62.0 ± 9.6	69.2 ± 8.4	59.8 ± 9.9

(Abbreviations: h, p1, and p2 refer to the mean empathy rating of the according condition, where h indicates data of healthy subjects, p1 indicates data of pre-treatment somatoform patients, and p2 indicates data of post-treatment somatoform patients; ± indicates the 95%-confidence-interval).

**Table 2:** **Effect of psychotherapy on hemodynamic responses—ROI based approach**.

**Region**		**Coordinates**			**fMRI contrast values**			**Psychotherapy effect**
		***x***	***y***	***z***	***h***	***p*1**	***p*2**	***p*2 > *p*1**
***All emotions ([“anger”+ “disgust” + “joy” + “neutral”] − “control”)***								
Right	parahippocampal gyrus	30	54	−3	−0.51 ± 1.77	−5.53 ± 2.25	−4.26 ± 2.60	*t*_(14)_ = 0.670; *p*_[one-tailed]_ = 0.257
Left	amygdala	−24	−3	−24	0.50 ± 0.87	−0.59 ± 1.54	−0.27 ± 1.47	*t*_(14)_ = 0.355; *p*_[one-tailed]_ = 0.364
***Anger (“anger” − “control”)***								
Left	Postcentral gyrus	−15	39	66	0.60 ± 0.70	−0.83 ± 0.83	0.41 ± 0.84	*t*_(14)_ = 2.042; *p*_[one-tailed]_ = 0.030^*^
Left	Superior temporal gyrus	−33	−15	−27	1.05 ± 0.60	−0.91 ± 1.03	0.23 ± 0.94	*t*_(14)_ = 1.899; *p*_[one-tailed]_ = 0.039^*^
Left	Parahippocampal gyrus	−33	18	−24	0.78 ± 0.60	−0.39 ± 0.52	0.19 ± 0.81	*t*_(14)_ = 1.914; *p*_[one-tailed]_ = 0.038^*^
Right	Parahippocampal gyrus	18	21	−15	1.20 ± 0.91	−0.92 ± 0.98	0.77 ± 0.63	*t*_(14)_ = 3.829; *p*_[one-tailed]_ < 0.001^***^^†^
Left	Posterior insula	−36	33	15	0.66 ± 0.50	−0.56 ± 0.62	0.12 ± 0.64	*t*_(14)_ = 2.151; *p*_[one-tailed]_ = 0.025^*^
Left	Amygdala	−21	−3	−21	1.24 ± 0.83	−0.57 ± 1.06	0.37 ± 0.77	*t*_(14)_ = 1.621; *p*_[one-tailed]_ = 0.064^(*)^
Left	Cerebellum	−36	81	−24	3.64 ± 1.34	0.97 ± 1.34	2.72 ± 1.40	*t*_(14)_ = 3.633; *p*_[one-tailed]_ = 0.001^**^^†^
***Joy (“joy” − “control”)***								
Right	Parahippocampal gyrus	30	54	−3	0.29 ± 0.42	−1.57 ± 0.74	−1.07 ± 0.77	*t*_(14)_ = 0.919; *p*_[one-tailed]_ = 0.187
Right	Cerebellum	33	84	−27	3.70 ± 1.40	0.31 ± 1.25	0.65 ± 1.43	*t*_(14)_ = 0.628; *p*_[one-tailed]_ = 0.270
Right	Cerebellum	21	87	−30	3.35 ± 1.44	0.47 ± 0.95	0.55 ± 1.01	*t*_(14)_ = 0.159; *p*_[one-tailed]_ = 0.438

In a previous study of our group, 12 regions of interest (ROIs) had shown significantly reduced modulation of hemodynamic responses during the same paradigm in pre-treatment somatoform disorder patients. Here, we investigated whether contrast values extracted from these ROIs showed a significant normalization after psychotherapy. In six ROIs, we found a significant enhancement of hemodynamic modulation after psychotherapy. After correction for multiple comparisons using a Bonferroni-correction, two ROIs (i.e., the right parahippocampal gyrus and the left cerebellum) showed a significant effect. (Abbreviations: x, y, and z refer to the Talairach coordinates of the regions; h, p1, and p2 refer to the contrast value of the according contrast, where h indicates data of healthy subjects, p1 indicates data of pre-treatment somatoform patients, and p2 indicates data of post-treatment somatoform patients; ± indicates the 95%-interval;

(*) : p < 0.1;

* : p < 0.05;

** : p < 0.01;

*** : p < 0.001;

† : p < 0.004, this indicates a significant effect after controlling for multiple comparisons using a Bonferroni-correction.)

**Table 3:** **Effect of psychotherapy on hemodynamic responses—voxel-wise whole brain analysis**.

**Region**		**Coordinates**			**Peak *t* value**	**Cluster size**	**FWE value**	**Effect**
		***x***	***y***	***z***				
***All emotions ([“anger”+ “disgust” + “joy” + “neutral”] − “control”)***								
No	Region
***Anger (“anger” − “control”)***								
Left	Parahippocampal gyrus	−25	41	−16	5.560	11	0.998	p2 > p1
Rightt	Parahippocampal gyrus	22	26	−16	6.130	11	0.998	p2 > p1
Left	Putamen	−26	−11	5	5.322	11	0.998	p2 < p1
***joy (“joy” − “control”)***								
Left	Inferior temporal gyrus	−53	−1	−32	6.173	12	0.960	p2 > p1

We implemented a voxel-wise whole brain analysis to identify brain regions with significant changes of hemodynamic modulation for the contrasts all emotions ([“anger”+ “disgust” + “joy” + “neutral”] − “control”), anger (“anger” − “control”), and joy (“joy” − “control”). (Abbreviations: x, y, and z refer to the Talairach coordinates of the center of mass of the regions; peak t value refers to the t value of the peak voxel in the cluster; cluster size refers to the number of voxels which survived threshold masking at p_[uncorrected]_ ≤ 0.001; FWE value describes the probability, that a cluster of the given size would appear as a false positive result in a contrast of the given smoothness; effect refers to an increase (p2 > p1) or a decrease (p2 < p1) of hemodynamic responses after psychotherapy.)

#### Control for potential effects of the psychotropic medication

Since 5 of the 15 patients were on psychotropic medication during either on one or both scanning sessions, we implemented two additional fMRI analyses (i.e., an additional ROI-based approach and an additional voxelwise approach) including only data of the 10 patients without medication. These results support the view that modulation of hemodynamic responses in the left superior temporal gyrus, right parahippocampal gyrus, left posterior insula, and left cerebellum (ROI-based approach), as well as in the in the bilateral parahippocampal gyrus, left putamen, and left inferior frontal gyrus (voxelwise approach) are not caused by the effects of psychotropic medication. Please see the Supplementary Material for a detailed presentation of the according results.

## Discussion

### Summary of findings

Somatoform disorder patients in a pre-treatment stage show diminished modulation of hemodynamic responses during emotional empathy in several brain areas, including the bilateral parahippocampal gyrus, left amygdala, left postcentral gyrus and others; this was reported in a previous paper by our group (de Greck et al., 2012). Here, we investigated whether brain activity normalized after multimodal psychodynamic psychotherapy. Psychotherapy was successful as demonstrated by a significant reduction of somatization symptoms (based on the SCL-90 somatization sub-scale), alexithymia symptoms (TAS-20), and depressive symptoms (BDI). In addition, psychotherapy led to significant reduction of error rates in an emotion recognition test (TAB).

The analysis of psychotherapy induced changes of brain activity was implemented using two approaches: a region of interest (ROI) based approach and a voxel-wise whole brain analysis. Both analyses came to the matching results that brain activity in the bilateral parahippocampal gyrus during empathy with anger normalized after psychotherapy. With regard to the ROI based analysis, we found that brain activity in almost all of those regions which had shown diminished modulation of hemodynamic responses in the pre-treatment stage for the contrast “empathy with anger” − “control” normalized after psychotherapy. Regions with a normalization after psychotherapy included the left postcentral gyrus, left superior temporal gyrus, left posterior insula, left amygdala (statistical trend), left cerebellum, and the above mentioned bilateral parahippocampal gyrus.

However, none of those regions which had shown diminished modulation of hemodynamic responses of pre-treatment somatoform patients during other contrasts (namely “empathy with all emotions” − “control,” and “empathy with joy” − “control”), showed a normalization of hemodynamic responses after psychotherapy.

With regard to the voxel-wise whole brain analysis, we found three regions, which had a significant change in neuronal activity after psychotherapy for the contrast “empathy with anger” - “control” (the bilateral parahippocampal gyrus, and the left putamen), and one region for the contrast “empathy with joy” − “control” (the left inferior temporal gyrus). Interestingly, the left putamen showed *less* modulation of hemodynamic responses after psychotherapy, whereas modulation was increased in all other regions. In addition, the left putamen was the only region in which reduction of hemodynamic responses was correlated with the reduction of somatic symptoms after psychotherapy (over all patients).

### Psychodynamic mechanisms in somatoform disorder

From a psychodynamic perspective, the development of somatoform symptoms can be understood as malfunctioning of secondary process mechanisms concerning the handling of emotional conflicts which leads to “resomatization” (i.e., the appearance of somatoform symptoms as “concomitants” or “equivalents” of affective tensions, Schur, 1955). In other words: unreleased affective tensions caused by unconscious emotional conflicts induce the corresponding somatic responses (which appear as somatic symptoms), whereas the corresponding affective component is repressed and can not be experienced (Lipowski, 1988; Hoffmann et al., 2004). In this regard, somatization (i.e., the development of medically unexplained somatic symptoms, Lipowski, 1988) is either seen as a defense mechanism itself (Bond et al., 1983; Lipowski, 1988), or as a correlate of immature defense styles (Nickel and Egle, 2006). In addition to emotional conflicts, the experience of overwhelmingly strong negative emotional reactions, which may occur for instance as a response to childhood adversities, can lead to a breakdown of secondary process thinking and a malfunction of mature defense mechanisms (Nickel and Egle, 2001). As a consequence, these patients are prone to develop reduced emotional awareness as correlate of the repressed emotional component, and somatic symptoms as equivalents of affective tensions (Krystal, 1997). Indeed, there is a strong link between childhood adversities and adult somatization (Spitzer et al., 2008).

### How does psychotherapy help patients with somatoform disorders?

Psychodynamic psychotherapy, as it was applied in our study, aims to increase the insight and acceptance of unconscious emotions, needs and conflicts (Blagys and Hilsenroth, 2002; Leichsenring, 2005), and to enable patients to gain “mastery over his or her repressed wishes, desires, fears, or anxieties” (Blagys and Hilsenroth, 2002). Regarding this, the development of more mature defense mechanisms and coping strategies is a core aim (Vaillant, 1977). Further treatment goals include the establishment of a psychosomatic disease model, the enhancement of affect differentiation, a better understanding of underlying stresses, and the reduction of medication abuse (Beutel et al., 2008). In the case of somatoform disorder, successful psychotherapy leads to enhanced secondary process thinking, more mature defense mechanisms, improved “desomatization”, and decreased somatic symptoms.

The reduction of somatic symptoms after psychotherapy in our study reflects a decreased use of somatization (i.e., improved “desomatization”). Improved emotion recognition after psychotherapy is probably caused by the uncovering of repressed emotional conflicts and emotional needs, which hampered emotion recognition in the pre-treatment stage.

### Neurophysiological mechanisms in somatoform disorder

The bilateral parahippocampal gyrus seems to play a key role in the affective dysfunctions of somatoform disorder (in particular in the processing of angry facial stimuli), since we found a significantly diminished modulation of hemodynamic responses in this area in the pre-treatment stage (de Greck et al., 2012) and a significant improvement after psychodynamic psychotherapy. Interestingly, the parahippocampal gyrus is involved in the recall of autobiographical memories (Maguire, 2001; Niki and Luo, 2002; Rekkas and Constable, 2005; Beni and Venneri, 2006), in particular the retrieval of emotional memories (Damasio et al., 2000), or emotional background informations (Smith et al., 2004; Sterpenich et al., 2006). In addition, the recall of conflictual memory content activates the parahippocampal gyrus (Loughead et al., 2010), and it is less activated during free associations to conflict-related themes (Schmeing et al., 2013).

### Affective processing in somatoform disorder and the effect of psychotherapy

High co-morbidity of somatoform disorder and alexithymia (i.e. diminished awareness of own and other's emotional processes) has been reported by various investigators (Bach and Bach, 1996; Bankier et al., 2001; Duddu et al., 2003; Grabe et al., 2004; Burba et al., 2006; Bailey and Henry, 2007; Mattila et al., 2008; Wood et al., 2009). From a psychodynamic point of view, the link between alexithymia and somatization is explained by a breakdown of secondary process coping with emotional conflicts, followed by the repression of the corresponding affective component and the experience of the somatic component as unexplained body symptoms (Schur, 1955; Lipowski, 1988; Hoffmann et al., 2004). Our data suggests that the parahippocampal gyrus, a region known for its involvement in the processing of autobiographic emotional memories, might be a neural key correlate of this process. In our study, we investigated brain activity during the intentional empathic processing of facial emotions. The process of intentional emotional empathy rests upon emotional sub processes which include emotional and cognitive empathy, emotion recognition, affective and cognitive mentalizing and the processing of autobiographical memory (Shamay-Tsoory, 2011). In order to empathize with somebody else, it is essential to generate a congruent emotional image of the target's emotional state within oneself (Preston and de Waal, 2002). Besides emotional contagion (Preston and de Waal, 2002; Shamay-Tsoory, 2011), this process relies on the recall of autobiographic memory traces (Damasio et al., 2000): If I want to understand from your face, what you feel, and intentionally want to share your emotional state, I may try to recall own memory traces which previously led to a similar emotional response in myself. This process involves the retrieval of emotional autobiographic memory and induces activity in the parahippocampal gyrus. In the case of somatoform disorder, however, it is assumed that overwhelmingly strong emotional conflicts led to a breakdown of emotional processing and a suppression of the corresponding memory traces, resulting in a diminished modulation of the parahippocampal gyrus during emotional empathy. Psychodynamic psychotherapy, however, can restore the retrieval of repressed autobiographic memories, which leads to increased modulation of the parahippocampal gyrus.

### Psychoanalytical neuroscience

We believe our study is a fine example, which demonstrates how neuroscience can benefit from psychoanalysis. Our main finding (i.e., pre-treatment somatoform disorder patients show diminished modulation of neuronal activity in the parahippocampal gyrus during the empathic processing of angry faces; this pattern normalizes after psychotherapy) fits well to psychoanalytical concepts of somatoform disorder. In fact, it is hard to explain our findings without referencing to psychodynamic concepts, which relate somatoform disorder to repressed emotional memories.

When it comes to the investigation of the neuronal underpinnings of complex emotional processing (and in particular its dysfunctions in psychosomatic diseases such as somatoform disorder) psychoanalytical concepts may provide a profound base to interpret neuroscientific findings.

## Limitations

Our psychotherapeutic intervention was “multimodal”, i.e., it included a variety of different therapeutic techniques. Hence, we can not conclude for sure that increased emotional insight in formerly repressed conflicts caused the reduction of symptoms and normalization of brain activity (and not for instance other therapeutic variables such as decreased depression).

Even more important, with the existing design, we can not for sure conclude that changes observed in the pre-post comparison are indeed caused by psychotherapy since they might be related to other factors, such as for instance retest effects, spontaneous remission, or in particular, regression to the mean effects (Barnett et al., 2005). To control for these potential confounds it would have been essential to scan a second control group consisting of somatoform patients, who would have been also scanned for two times without participation in psychotherapy.

However, the fact that we found reduced neuronal activity in the *bilateral* parahippocampal gyrus in the pre-treatment stage, and normalized activity in the *bilateral* parahippocampal gyrus in the post-treatment stage, supports the view that these activations are not related to regression to the mean effects (because it would be rather improbable that regression to the mean effects lead to bilateral effects in the same brain region).

## Conclusion

Our results are in accordance with the conclusion that the repression of emotional memories, which occurs in somatoform disorder in order to defend against overwhelmingly strong emotions, can neurophysiologically be understood in terms of diminished activation of the parahippocampal gyrus. Psychotherapeutic measures aim to increase emotional insight and to accept repressed feelings and emotional memories. After psychotherapy, somatoform patients reported less symptoms and showed stronger neuronal activity in the parahippocampal gyrus. Our results support the assumption that increased access to repressed emotional memories is related to increased neuronal activity of the parahippocampal gyrus.

### Conflict of interest statement

The authors declare that the research was conducted in the absence of any commercial or financial relationships that could be construed as a potential conflict of interest.
