# Supplementary material for: Changes in brain activity of somatoform disorder patients during emotional empathy after multimodal psychodynamic psychotherapy
Source: Front Hum Neurosci. 2013 Aug 16;7:410. doi: 10.3389/fnhum.2013.00410 (PMC3744921; doi:10.3389/fnhum.2013.00410)
Supplement: Supplementary file 1 [file DataSheet1.ZIP › Supplementary material.pdf]

Multimodal psychodynamic psychotherapy modulates brain activity of somatoform disorder patients during emotional empathy

Supplementary Material

A. Description of psychotherapeutic techniques

Psychodynamic sychotherapy was applied in a standardized multimodal inpatient setting which was recently explained (de Greck et al., 2011; Grabe et al., 2008; Haase et al., 2008; Huber et al., 2009). It included the following therapeutic measures:

A.1. Psychodynamic individual psychotherapy

Psychodynamic individual psychotherapy was limited to 100 minutes per week. Aims of psychodynamic individual psychotherapy were the verbalization of emotional and interpersonal problems, and the enhancement of unconscious emotions, needs and conflicts (Blagys and Hilsenroth, 2002; Grabe et al., 2008; Leichsenring, 2005), in order to enable the patient to utilize a broader spectrum of coping strategies (Vaillant, 1977). Within this setting, the psychotherapist provided a space for the patient to address his problems. The psychotherapists interpreted resistances, confronted the patient with his behavior, and detected connections between the patient's actual behavior / symptoms and his biography.

A.2. Psychodynamic group psychotherapy

Psychodynamic group therapy was implemented for between 120 and 270 minutes per week. Aims of psychodynamic group psychotherapy were the verbalization of individual and interpersonal problems. The group situation enabled a group dynamic, which brought forth actual interpersonal problems with their according emotional dynamic. Participants of the group therapy gave mutual feedback about the way they perceived the appearance and the behavior of their copatients. This setting provided the possibility for each participant to reflect about himself and his behavioral patterns in social situations. The psychotherapists focused on the observation and interpretation of emotional dynamics, emotional atmosphere, and conflicts.

A.3. Music therapy

Music therapy was conducted for approximately 3 x 90 minutes per week. Aims of music therapy were the non-verbal communication the patients' actual emotional conflicts and moods. Music therapy was performed in a group setting. At the beginning of each session, patients were given opportunity, to speak about their current issues. This was followed by a musical improvisation part, during which patients played different instruments (for instance xylophone, drums, triangle), whilst the music therapist accompanied on the piano. The improvisation was recorded and played to the patients after the improvisation. Finally, the music therapist led a reflection round.

A.4. Communicative movement therapy

Communicative movement therapy was conducted for approximately 1 x 60 minutes per week. It included different exercises to increase body awareness (for instance walking through warm sand, grass, etc.), awareness of non-verbal social interactions ("How do I argue with others?", "How can I accomplish my aims?"), or awareness of emotions in the context of distance and proximity. The therapist was responsible for the thematic content of each session. He observed, gave feedback and led through a final reflection round.

A.5. Art therapy

Art therapy was conducted for approximately 2 x 90 minutes per week. Art therapy aimed to increase the access to unconscious feelings by means of a non-verbal, creative activity. The art therapist provided the thematic content of each session (for instance ”the group and me”) and led through a closing feedback- and reflection round.

A.6. Social therapy

Social therapy was conducted for approximately 1 x 60 and 1 x 90 minutes per week. Aims of social therapy were the discussion of social issues concering family, work, etc.

A.7. Relaxation methods

Patients were encouraged to learn and engage in the following relaxation techniques: autogenic training and progressive muscle relaxation. Autogenic training was practised for approximately 2 x 30 minutes per week. It was conducted in a group setting according to the established standards. Progressive muscle relaxation was practised for approximately 2 x 30 minutes per week. It was conducted in a group setting according to the established standard by Jacobsen.

B. Confirmatory analyses

To control for medication effects, we implemented two additional statistical analyses including only the fMRI data of the 10 patients without psychotropic medication.

As demonstrated in Table B.1, the results of the ROI-based analysis support the conclusion that enhancement of hemodynamic modulation in the left superior temporal gyrus, right parahippocampal gyrus, left posterior insula, and left cerebellum is not caused by the effects of psychotropic medication.

In addition (as demonstrated in Table B.2), the confirmatory analysis based on a voxel-wise whole brain approach support the conclusion, that the enhancement of hemodynamic modulation in the bilateral parahippocampal gyrus, left putamen, and left inferior frontal gyrus is not caused by the effects of psychotropic medication.

Table B.1

**Region**		**coordinates** x	y	z	**fMRI contrast values** p1	p2	**psychotherapy effect** p2 > p1 (n=10)
					all emotions ([‘anger’+ ‘disgust’ + ‘joy’ + ‘neutral’] - ‘control’)		
right	parahippocampal gyrus	30	54	-3	-4.16 ± 2.49	-5.42 ± 3.60	$t(9) = -0.586$; p2 < p1
left	amygdala	-24	-3	-24	-0.44 ± 1.99	-0.61 ± 2.02	$t(9) = -0.145$; p2 < p1
					anger (‘anger’ - ‘control’)		
left	postcentral gyrus	-15	39	66	0.31 ± 0.85	0.00 ± 0.84	$t(9) = 0.832$; $p_{[one-tailed]} = 0.214$
left	superior temporal gyrus	-33	-15	-27	-0.88 ± 1.41	0.45 ± 0.67	$t(9) = 1.732$; $p_{[one-tailed]} = 0.056^{(*)}$
left	parahippocampal gyrus	-33	18	-24	-0.25 ± 0.72	0.17 ± 1.12	$t(9) = 1.289$; $p_{[one-tailed]} = 0.115$
right	parahippocampal gyrus	18	21	-15	-1.01 ± 1.35	0.41 ± 0.61	$t(9) = 2.288$; $p_{[one-tailed]} = 0.024$*
left	posterior insula	-36	33	15	-0.63 ± -0.84	-0.03 ± 0.68	$t(9) = 1.626$; $p_{[one-tailed]} = 0.069^{(*)}$
left	amygdala	-21	-3	-21	-0.32 ± 0.97	-0.08 ± 0.85	$t(9) = 0.390$; $p_{[one-tailed]} = 0.353$
left	cerebellum	-36	81	-24	0.95 ± 1.57	2.28 ± 1.69	$t(9) = 2.099$; $p_{[one-tailed]} = 0.033$*
					joy (‘joy’ - ‘control’)		
right	parahippocampal gyrus	30	54	-3	-1.25 ± 1.00	-1.32 ± 1.08	$t(9) = -0.104$; p2 < p1
right	cerebellum	33	84	-27	0.40 ± 1.53	1.15 ± 1.77	$t(9) = 1.193$; $p_{[one-tailed]} = 0.132$
right	cerebellum	21	87	-30	0.12 ± 1.12	0.54 ± 1.38	$t(9) = 1.024$; $p_{[one-tailed]} = 0.166$

Table B.1: Effect of psychotherapy on hemodynamic responses - ROI based approach - controlling for medication effects
The table is a complement to to Table 2, including only those patients, who were without medication during both scanning sessions. Despite the lower statistical power of the smaller group, we found two ROIs with a significant enhancement of hemodynamic modulation after psychotherapy (and two additional ROIs revealing a statistical trend). These data confirm the conclusion that enhancement of hemodynamic modulation in the left superior temporal gyrus, right parahippocampal gyrus, left posterior insula, and left cerebellum is not caused by the effects of psychotropic medication.

(Abbreviations: x, y, and z refer to the Talairach coordinates of the regions; p1, and p2 refer to the contrast value of the according contrast, where p1 indicates data of pre-treatment somatoform patients, and p2 indicates data of post-treatment somatoform patients; ± indicates the 95%-interval; $^{(*)}$: p<0.1; *: p<0.05)

Table B.2

	Region	coordinates x	y	z	peak t value	effect
	all emotions ([‘anger’+ ‘disgust’ + ‘joy’ + ‘neutral’] - ‘control’)					
no	Region					
	anger (‘anger’ - ‘control’)					
left	parahippocampal gyrus	-25	41	-16	1.711	p2>p1
left	parahippocampal gyrus[1]	-24	39	-15	10.067	p2>p1
right	parahippocampal gyrus	22	26	-16	3.213	p2>p1
right	parahippocampal gyrus[1]	24	27	-15	4.430	p2>p1
left	putamen	-26	-11	5	-4.232	p2<p1
left	putamen[1]	-28	-12	3	-4.838	p2<p1
	joy (‘joy’ - ‘control’)					
left	inferior temporal gyrus	-53	-1	-32	2.445	p2>p1
left	inferior temporal gyrus[1]	-49	-6	-37	5.108	p2>p1

Table B.2: Effect of psychotherapy on hemodynamic responses - voxel-wise whole brain analysis - controlling for medication effects

The table is a complement to to Table 3, including only those patients, who were without medication during both scanning sessions. All four regions listed in Table 3 show significant activity in group analyses including only those patients without psychotropic medication. These data confirm the conclusion that enhancement of hemodynamic modulation in the bilateral parahippocampal gyrus, left putamen, and left inferior frontal gyurs is not caused by the effects of psychotropic medication.

(Abbreviations: x, y, and z refer to the Talairach coordinates of the center of mass of the regions found in the statistical analyses including all 15 subjects; peak t value refers to the t value of the peak voxel in the cluster; effect refers to an increase (p2>p1) or a decrease (p2<p1) of hemodynamic responses after psychotherapy. [1]: these voxels are the peak voxels found in the statistical analyses including the 10 unmedicated patients.)

C. References

Blagys, M. D. and Hilsenroth, M. J. (2002). Distinctive activities of cognitive-behavioral therapy. a review of the comparative psychotherapy process literature. *Clin. Psychol. Rev.* 22, 671–706.

de Greck, M., Scheidt, L., Bölter, A. F., Frommer, J., Ulrich, C., Stockum, E., Enzi, B., Tempelmann, C., Hoffmann, T., and Northoff, G. (2011). Multimodal psychodynamic psychotherapy induces normalization of reward related activity in somatoform disorder. *World J. Biol. Psychiat.* 12, 296–308.

Grabe, H. J., Frommer, J., Ankerhold, A., Ulrich, C., Groger, R., Franke, G. H., Barnow, S., Freyberger, H. J., and Spitzer, C. (2008). Alexithymia and outcome in psychotherapy. *Psychother. Psychosom.* 77, 189–194.

Haase, M., Frommer, J., Franke, G. H., Hoffmann, T., Schulze-Muetzel, J., Jäger, S., Grabe, H. J., Spitzer, C., and Schmitz, N. (2008). From symptom relief to interpersonal change: Treatment outcome and effectiveness in inpatient psychotherapy. *Psychotherapy Res.* 18, 615–624.

Huber, D., Albrecht, C., Hautum, A., Henrich, G., and Klug, G. (2009). [effectiveness of inpatient psychodynamic psychotherapy: a follow-up study]. *Z. Psychosom. Med. Psychother.* 55, 189–199.

Leichsenring, F. (2005). Are psychodynamic and psychoanalytic therapies effective?: A review of empirical data. *Int. J. Psychoanal.* 86, 841–868.

Vaillant, G. E. (1977). *Adaptation to Life*. Boston, MA, USA: Harvard University Press.
